# Candidemia on presentation to the hospital: development and validation of a risk score

**DOI:** 10.1186/cc8110

**Published:** 2009-09-29

**Authors:** Andrew F Shorr, Ying P Tabak, Richard S Johannes, Xiaowu Sun, James Spalding, Marin H Kollef

**Affiliations:** 1 Pulmonary and Critical Care Medicine Service, Washington Hospital Center, Washington, DC 20010, USA; 2 Clinical Research, MedMined™ Services, CareFusion, 400 Nickerson Road, Marlborough, MA 01752, USA; 3 Division of Gastroenterology, Harvard Medical School and Brigham and Women's Hospital, Boston, MA 02115, USA; 4 Health Economics & Outcomes Research, Astellas Pharma US Inc., Three Parkway North, Deerfield, IL 60015, USA; 5 Pulmonary and Critical Care Division, Washington University School of Medicine, 660 South Euclid Ave, St. Louis, MO 63110, USA

## Abstract

**Introduction:**

Candidemia results in substantial morbidity and mortality, especially if initial antifungal therapy is delayed or is inappropriate; however, candidemia is difficult to diagnose because of its nonspecific presentation.

**Methods:**

To develop a risk score for identifying hospitalized patients with candidemia, we performed a retrospective analysis of a large database of 176 acute-care hospitals in the United States. We studied 64,019 patients with bloodstream infection (BSI) on presentation from 2000 through 2005 (derivation cohort) and 24,685 from 2006 to 2007 (validation cohort). We used recursive partitioning (RPART) to identify the best discriminators for *Candida *as the cause of BSI. We compared three sets of models (equal-weight, unequal-weight, vs full model with additional variables from logistic regression model) for sensitivity analysis.

**Results:**

The RPART identified 6 variables as the best discriminators: age < 65 years, temperature ≤ 98°F or severe altered mental status, cachexia, previous hospitalization within 30 days, admitted from other healthcare facility, and need for mechanical ventilation. The prevalence for patients presented with 0 through 6 risk factors in the derivation cohort was 28.7%, 38.8%, 21.8%, 8.3%, 2.1%, 0.3%, and < 0.1% respectively. The corresponding candidemia rates were 0.4% (69/18,355), 0.8% (196/24,811), 1.6% (229/13,984), 3.2% (168/5,330), 4.2% (58/1,371), 9.6% (15/157), and 27.3% (3/11) respectively (*P *< 0.0001). Findings were similar in the validation cohort (*P *< 0.0001). The equal-weight risk score model, which signed 1 point to each risk factor, yielded good discrimination in both cohorts with areas under the receiver operating curve (AUROCs) of 0.70 versus 0.71 (derivation versus validation). AUROC values were similar for the unequal-weight model, which signed different weight to each risk factor based on multivariable logistic regression coefficient, (AUROCs, 0.70-0.72). Both equal-weight and unequal-weight models were well calibrated (all Hosmer-Lemshow *P *> 0.10, indicating predicted and observed candidemia rates did not differ significant across the 7 risk stratus). The full model with 16 risk factors had slightly higher AUROCs (0.74 versus 0.73 for derivation versus validation); however, 7 variables were no longer significant in the recalibrated model for the validation cohort, indicating that the additional items did not materially enhance the model.

**Conclusions:**

A simple equal-weight risk score differentiated patients' risk for candidemia in a graded fashion upon hospital presentation.

## Introduction

Candidemia represents the fourth most common type of hospital-acquired bloodstream infection (BSI) [1-3]. More importantly, candidemia results in substantial morbidity [4-8] and mortality [7-10], especially if initial antifungal therapy is delayed or is not appropriate [5,11,12]. Delaying therapy by as little as 12 hours after obtaining a blood culture can double the risk of death [11]. Therefore, prompt initiation of antifungal therapy is a key determinant of outcome. Complicating efforts to identify subjects at risk for candidemia is the expansion of healthcare delivery beyond the hospital and the evolving recognition of distinct healthcare-associated infection syndromes [13-15], *Candida *may now represent a cause of BSI in patients presenting to the hospital [8].

Given the need to ensure appropriate and timely antifungal therapy and to optimally separate patients at low risk for candidemia from those at high risk, some form of risk stratification for candidemia becomes imperative. This is particularly true for those with candidemia on admission to the hospital because clinicians rarely consider this diagnosis in this setting. The nonspecific signs and symptoms of candidemia further frustrate efforts at early patient identification [16]. Although biomarkers such as (1→3)-β-D-glucan are being investigated [17], they are not likely to prove useful in patients presenting to the hospital. The traditional approach to assessing the probability of *Candida *as a cause of nosocomial BSI has relied upon assessing the number and type of risk factors (e.g., corticosteroid therapy, total parenteral nutrition); however, this strategy has proven to have little utility in critically ill patients and proposed schema for risk stratification have yet to be well validated.

We hypothesized that, despite frustration with clinical risk stratification paradigms for inpatients, assessment of select characteristics could identify patients presenting to the hospital who are at heightened risk for candidemia. We further theorized that these select characteristics could be used to develop a prediction rule to indicate which patients are likely to have BSI due to *Candida *as opposed to a bacterial pathogen.

## Materials and methods

### Design

To develop a clinical risk score for identifying patients with BSI likely to be caused by *Candida *spp. upon hospital presentation, we performed a retrospective analysis of patients discharged from 176 acute-care hospitals in the United States from 2000 to 2005. We validated the risk score with discharge data from the same hospitals from 2006 to 2007.

### Data

We used the CareFusion Outcomes Research Database (Clinical Research Services, CareFusion, Marlborough, MA, USA), which has been described previously [14,15,18-22]. The database comprises acute-care admissions at participating hospitals, including electronically imported or manually abstracted demographic, clinical (e.g., comorbidities, vital signs, laboratory values, other clinical findings), and administrative data (e.g., diagnosis). The underlying data for this study are a limited data set with all patient specific information anonymized. This study was reviewed and approved by the New England Institutional Review Board/Human Subjects Research Committee (Wellesley, MA, USA). It was conducted in compliance with US federal regulations, Health Insurance Portability and Accountability Act, and the Helsinki Declaration.

The outcome for deriving the risk score was BSI due to *Candida *spp. as defined by the presence of a blood culture positive for *Candida*, and a concomitant primary or secondary diagnostic code (*International Classification of Diseases*, Ninth Revision, Clinical Modification (*ICD*-9-CM)) indicative of candidemia. We required that blood samples had been drawn within one day before or within two days after hospital admission. This database undergoes multiple quality assurance assessments with periodic data auditing. In order to limit coding bias we require concomitant presence of an *ICD*-9 code for candidemia and a positive blood culture. We did not explore other forms of invasive candidiasis.

### Variables

Candidate variables were selected *a priori *based on their biologic plausibility of explaining risk for candidemia. Specifically, we explored demographic factors (age, gender), vital signs, mental status, laboratory test results, and underlying comorbid conditions. Vital signs included pulse, blood pressure, temperature, and respiratory rate. Altered mental status (AMS) was defined by a Glasgow Coma Scale (GCS) score of 10 to 14 or disoriented/lethargy (mild AMS); GCS 5 to 9 (moderate AMS); GCS less than 5 or a designation of 'coma' as charted by a physician (severe AMS). Laboratory testing included serum albumin; blood urea nitrogen (BUN); creatinine; sodium; potassium; glucose; hemoglobin; white blood cell count; and other routine chemistry, hematology, blood gas, and metabolic results. Comorbid conditions included cachexia (*ICD*-9 secondary diagnosis code), history of malignancy, diabetes, chronic heart failure, and other chronic conditions abstracted through chart review or secondary *ICD*-9 diagnostic codes. In addition, we explored variables pertinent to candidemia and were available in the data base, such as hemodialysis, immunosuppressive medication, previous hospitalization within 30 days, transfer from another healthcare facility, and mechanical ventilation on admission. Certain patient characteristics were not available in this database. For example, utilization of parenteral nutrition outside the hospital and prior antibiotic exposure are not recorded in this database. Vital signs and other patient-specific characteristics were obtained within one day of admission. For each vital sign and laboratory test result, we used the worst value obtained in the emergency department or, if not available, on the day of admission.

### Risk score development

To identify risk factors that optimally separate patients at low risk for candidemia from those at high risk, we used a recursive partition (RPART) approach [23]. Also referred to as classification and regression tree analysis [24], RPART has been used to derive prediction rules for acute chest pain [25], heart failure [26], and other conditions [27,28]. RPART first identifies the variable with the highest discrimination for the outcome of interest (node) and then repeats the process to partition subsequent nodes. RPART yields a tree-like algorithm with numerous nodes. To further improve ease of use, we simplified the algorithm based on the number of risk factors present, giving equal weight (one point) to each risk factor identified in by the RPART (equal-weight risk score).

### Risk score validation

To validate the model, we applied the derived risk score to patients in the validation cohort. We compared the between-cohort distribution of candidemia prevalence by risk score strata for the validation cohort with that from the derivation cohort and performed the Cochrane-Armitage test to assess trend [29]. We used the area under the receiver operating curve (AUROC) to assess the discrimination of the model and Hosmer-Lemshow test to assess model calibration. A higher value for the Hosmer-Lemshow test indicates better model fit.

### Sensitivity analysis

Using AUROC and Hosmer-Lemshow goodness-of-fit statistics, we compared the discrimination and calibration of the simpler versus more complex models. Specifically, we fit three sets of logistic regression models. The first was the equal-weight risk-score model, which was a univariate logistic regression model using a single continuous variable of the number of risk factors present (ranging from 0 to 6). This model gave the same weight for each risk factor present. The second was the unequal-weight risk factor model, which was a multivariable logistic regression model using each of the same variables in the equal-weight risk score as covariates. The unequal weight model assigned different weights for each variable per multivariable logistic regression coefficients. The third model was the full risk factor model, which was generated from a stepwise multivariable logistic regression analysis with additional variables retained in the model that were significant (*P *< 0.05).

Statistical analyses were performed using Statistical Analysis Software (SAS, version 9.01; SAS Institute Inc., Cary, NC, USA). Two-sided *P *values < 0.05 were considered statistically significant.

## Results

### Baseline characteristics of derivation and validation cohorts

The derivation cohort included 64,019 admissions and the validation cohort included 24,685 (Table 1) [see Additional data file 1]. Many between-cohort differences in demographics, laboratory findings, vital signs, comorbidities, and other variables were statistically significant. For example, the derivation cohort had a smaller proportion of patients aged 64 years or younger, smaller proportion of men, and higher in-hospital mortality. Approximately 10% of patients needed mechanical ventilation on admission, including 9.2% of those in the derivation cohort and 10.9% of those in the validation cohort. Among patients needing mechanical ventilation, candidemia occurred in 2.3% of those in the derivation cohort and in 3.1% of those in the validation cohort (Table 2) [see Additional data file 2].

**Table 1:** Patient characteristics by cohort

	Number of admissions (% of total)		
		
Characteristic	Derivation cohort (n = 64,019)	Validation cohort (n = 24,685)	*P *value
**Candidemia **	738 (1.2)	321 (1.3)	0.0697
**Demographics**			
Age < 65 years	19,523 (30.5)	8403 (34.0)	< 0.0001
Men	29,845 (46.6)	12,090 (49.0)	< 0.0001
Mortality	9664 (15.1)	3173 (12.9)	< 0.0001
**Laboratory findings**			
Albumin ≤ 1.8 g/dL	2704 (4.2)	1278 (5.2)	< 0.0001
Albumin 1.9--2.2 g/dL	3800 (5.9)	1757 (7.1)	< 0.0001
Arterial pH ≤ 7.36	5913 (9.2)	2684 (10.9)	< 0.0001
Potassium > 5.6 mEq/dL	3133 (4.9)	1182 (4.8)	0.5150
Sodium > 145 mEq/dL	3600 (5.6)	1206 (4.9)	< 0.0001
Bands > 32%	5345 (8.4)	1813 (7.4)	< 0.0001
White blood cells > 27,000/mm^3^	5096 (8.0)	1980 (8.0)	0.7604
**Vital signs and mental status**			
Temperature ≤ 98°F	16,917 (26.4)	4780 (19.4)	< 0.0001
Severe altered mental status^*a*^	6301 (9.8)	2255 (9.1)	0.0014
Temperature ≤ 98°F or severe altered mental status	21,140 (33.0)	6454 (26.1)	< 0.0001
**History and severe comorbidities**			
Metastatic cancer	3245 (5.1)	1262 (5.1)	0.7910
Tumor	2693 (4.2)	1136 (4.6)	0.0094
Cachexia^*b*^	4549 (7.1)	2392 (9.7)	< 0.0001
**Other variables**			
Prior-admission within 30 days	11,215 (17.5)	4603 (18.7)	< 0.0001
Admitted from other healthcare facility	12,813 (20.0)	5581 (22.6)	< 0.0001
Mechanical ventilation at admission	5864 (9.2)	2695 (10.9)	< 0.0001

^*a *^Severe altered mental status defined as Glasgow Coma Scale less than 5 or a designation of coma by a physician.

^*b *^Cachexia defined by *ICD*-9 secondary diagnosis code.

### Derivation and validation of candidemia risk score

Univariate analysis revealed that the following variables were associated with candidemia: age younger than 65 years; cachexia; deranged albumin, arterial pH, and electrolytes; temperature of 98°F or less, or severe altered mental status; previous hospitalization within 30 days; admitted from other healthcare facility; and mechanical ventilation at admission (all *P *≤ 0.001; Table 2). These associations were similar in the derivation and validation cohorts.

**Table 2:** Univariate analysis of variables associated with candidemia

	Derivation cohort (n = 64,019)		Validation cohort (n = 24,685)	
	
Variable	Number of candidemia/Number of cases in the row (%)	*P *Value	Number of candidemia/Number of cases in the row (%)	*P *Value
**Candidemia cases/Total cases**	738/64,019 (1.2)		321/24,685 (1.3)	0.0697
**Demographics**				
Age < 65 years	331/19,523 (1.7)	< 0.0001	137/8403 (1.6)	0.0010
Men	377/29,845 (1.3)	0.0158	170/12,090 (1.4)	0.1508
**Laboratory findings**				
Albumin ≤ 1.8 g/dL	83/2704 (3.1)	< 0.0001	40/1278 (3.1)	< 0.0001
Albumin 1.9--2.2 g/dL	75/3800 (2.0)	< 0.0001	45/1757 (2.6)	< 0.0001
Arterial pH ≤ 7.36	130/5913 (2.2)	< 0.0001	61/2684 (2.3)	< 0.0001
Potassium > 5.6 mEq/dL	80/3133 (2.6)	< 0.0001	34/1182 (2.9)	< 0.0001
Sodium > 145 mEq/dL	74/3600 (2.1)	< 0.0001	34/1206 (2.8)	< 0.0001
Bands > 32%	50/5345 (0.9)	0.1239	26/1813 (1.4)	0.6021
White blood cells > 27,000/mm^3^	54/5096 (1.1)	0.5840	35/1980 (1.8)	0.0557
**Vital signs and mental status**				
Temperature ≤ 98°F	253/16,917 (1.5)	< 0.0001	99/4780 (2.1)	< 0.0001
Severe altered mental status^*a*^	135/6301 (2.1)	< 0.0001	51/2255 (2.3)	< 0.0001
Temperature ≤ 98°F or severe altered mental status^*a*^	336/21,140 (1.6)	< 0.0001	129/6454 (2.0)	< 0.0001
**History and severe comorbidities**				
Metastatic cancer	62/3245 (1.9)	0.0001	25/1262 (2.0)	0.0285
Tumor	41/2693 (1.5)	0.0786	19/1136 (1.7)	0.2570
Cachexia^*b*^	123/4549 (2.7)	< 0.0001	70/2392 (2.9)	< 0.0001
**Other variables**				
Pre-admission within 30 days	276/11,215 (2.5)	< 0.0001	125/4603 (2.7)	< 0.0001
Admitted from other healthcare facility	281/12,813 (2.2)	< 0.0001	133/5581 (2.4)	< 0.0001
Mechanical ventilation at admission	136/5864 (2.3)	< 0.0001	84/2695 (3.1)	< 0.0001

^*a *^Severe altered mental status defined as Glasgow Coma Scale less than 5 or a designation of coma by a physician.

^*b *^Cachexia defined by *ICD*-9 secondary diagnosis code.

**Figure 1:**
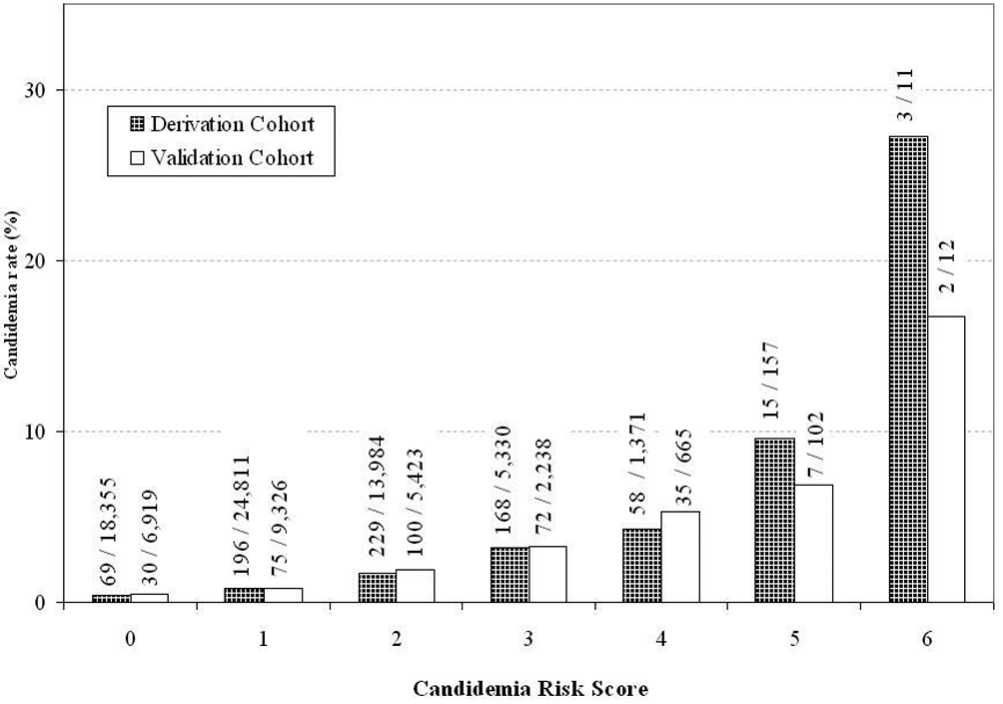
**Distribution of overall cases and Candidemia cases by the equal-weight Candidemia Risk Score**.

**Figure 2:**
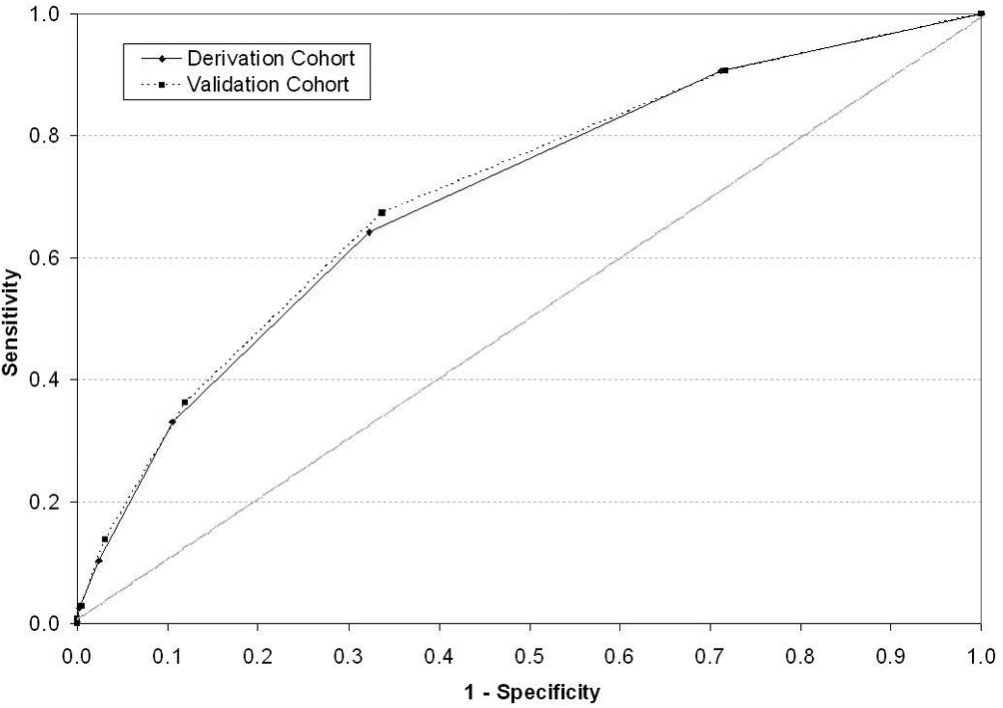
**Receiver operating characteristics curves for the equal-weight Candidemia Risk Score by cohort**. The area under the receiver operating curve was 0.70 for the derivation cohort and 0.71 for the validation cohort.

RPART revealed that the six best discriminators for candidemia were age younger than 65 years, temperature of 98°F or less, or severe altered mental status, cachexia, previous hospitalization within 30 days, admitted from other healthcare facility, and mechanical ventilation at admission. The prevalence for patients presented with 0 through to 6 risk factors in the derivation cohort was 28.7%, 38.8%, 21.8%, 8.3%, 2.1%, 0.3%, and less than 0.1%, respectively. The corresponding candidemia rates were 0.4% (69/18,355), 0.8% (196/24,811), 1.6% (229/13,984), 3.2% (168/5330), 4.2% (58/1371), 9.6% (15/157), and 27.3% (3/11), respectively (*P *< 0.0001). Findings were similar in the validation cohort (*P *< 0.0001; Figure 1). The Cochrane-Armitage test for trend was significant (*P *< 0.0001), confirming graded risk of candidemia with increased number of risk factors. Findings were similar in the validation cohort. The equal weight risk-score model provided good discrimination as demonstrated by the AUROC of 0.70 for the derivation cohort and 0.71 for the validation cohort (Figure 2).

In the derivation cohort, an overall score of 1 or more had an sensitivity of 90.7% and a negative predictive value (NPV) of 99.6% for the presence of candidemia. The specificity was more limited at 28.9%. The negative predictive value of each total point score remained above 99% so long as the number of risk factors presented remained less than 3. These findings were similar in the validation cohort. In other words, a low score nearly excluded the likelihood of candidemia. In patients with a score of zero, who account for nearly 30% of all subjects evaluated, there were very few cases of candidemia, with a NPV of 99.6%.

### Sensitivity analysis

The equal-weight risk model was associated with discrimination similar to that of the unequal-weight model (Table 3). The AUROCs (95% confidence intervals) for the equal-weight risk-score model were 0.70 (0.68 to 0.72) for the derivation cohort and 0.71 (0.68 to 0.74) for the validation cohort. The corresponding values for the unequal-weight model were 0.71 (0.70 to 0.73) and 0.72 (0.69 to 0.75). The full model with 16 risk factors was associated with slightly higher discrimination in both cohorts, with corresponding values of 0.74 (0.72 to 0.76) and 0.73 (0.70 to 0.76). Seven variables in 16-risk factor model, however, were not significant in the recalibrated model for the validation cohort, suggesting that using the additional covariates did not materially enhance the model.

**Table 3:** Sensitivity analysis of models for predicting candidemia

	Derivation cohort		Validation cohort	
	
Variable	OR (95% CI)	*P *value^*a*^	OR (95% CI)	*P *value
**Equal weight risk-score model**	**AUROC^*b *^= 0.70; H-L *P *= 0.39**		**AUROC = 0.71; H-L *P *= 0.34**	
Number of risk factors present (0--6)	1.93 (1.81--2.04)	< 0.0001	1.89 (1.73--2.06)	< 0.0001
**Unequal weight risk model**	**AUROC = 0.71; H-L *P *= 0.47 **		**AUROC = 0.72; H-L *P *= 0.66**	
Age < 65 years	2.08 (1.79--2.41)	< 0.0001	1.53 (1.21--1.92)	0.0003
Temperature ≤ 98°F or severe altered mental status^*c*^	1.43 (1.23--1.66)	< 0.0001	1.43 (1.13--1.81)	0.0030
Cachexia^*d*^	2.16 (1.77--2.64)	< 0.0001	2.01 (1.53--2.65)	< 0.0001
Prior admission within 30 days	2.54 (2.18--2.96)	< 0.0001	2.50 (1.99--3.15)	< 0.0001
Admitted from other health care facility	2.28 (1.95--2.66)	< 0.0001	2.06 (1.63--2.60)	< 0.0001
Mechanical ventilation at admission	1.56 (1.28--1.90)	< 0.0001	2.07 (1.58--2.71)	< 0.0001
**Full risk model (16 risk factors)**	**AUROC = 0.74; H-L *P *= 0.02 **		**AUROC = 0.73; H-L *P *= 0.74**	
Age < 65 years	2.03 (1.75--2.37)	< 0.0001	1.52 (1.21--1.92)	0.0004
Admitted from other health care facility	2.27 (1.93--2.65)	< 0.0001	2.06 (1.63--2.61)	< 0.0001
Mechanical ventilation at admission	1.28 (1.02--1.61)	0.0368	2.03 (1.48--2.78)	< 0.0001
Metastatic cancer	1.57 (1.20--2.05)	0.0011	1.43 (0.94--2.18)	0.0994
Tumor	1.47 (1.06--2.02)	0.0202	1.36 (0.85--2.19)	0.2008
Cachexia	1.99 (1.62--2.43)	< 0.0001	1.82 (1.38--2.41)	< 0.0001
Pre-admission within 30 days	2.41 (2.07--2.81)	< 0.0001	2.35 (1.86--2.97)	< 0.0001
Albumin ≤ 1.8 g/dL	1.70 (1.33--2.18)	< 0.0001	1.48 (1.03--2.13)	0.0329
Albumin 1.9--2.2 g/dL	1.35 (1.05--1.73)	0.0184	1.59 (1.14--2.22)	0.0061
Potassium > 5.6 mEq/dL	1.61 (1.26--2.06)	0.0002	1.42 (0.97--2.08)	0.0747
Sodium > 145 mEq/dL	1.30 (1.00--1.68)	0.047	1.51 (1.03--2.20)	0.0345
Arterial pH < 7.36	1.38 (1.10--1.74)	0.0058	1.00 (0.71--1.41)	0.9896
Bands > 32%	0.66 (0.49--0.88)	0.0051	0.87 (0.57--1.31)	0.4991
White blood cells > 27,000/mm^3^	0.69 (0.52--0.92)	0.0105	1.09 (0.76--1.57)	0.6296
Temperature ≤ 98°F	1.22 (1.04--1.43)	0.0130	1.52 (1.19--1.95)	0.0009
Severe altered mental status	1.26 (1.01--1.56)	0.0388	0.94 (0.67--1.33)	0.7403

AUROC = area under receiver operating curve; CI = confidence interval; H-L = Hosmer-Lemshow chi-squared test; OR = odds ratio.

^*a *^H-L *P *values for the three models were determined by Hosmer-Lemshow chi-squared test in which *P *> 0.05 indicates good model calibration of predicted vs observed candidemia across low- and high-risk deciles. *P *values for each variable within the models were determined by each logistic regression model in which a significant value indicates increased risk for candidemia.

^*b *^AUROC ≥ 0.70 indicates good model discrimination.

^*c *^Severe altered mental status defined by Glasgow Coma Scale less than 5 or a designation of coma by a physician.

^*d *^Cachexia defined by *ICD*-9 secondary diagnosis code.

Both the equal and unequal weight models provided good calibration of predicted versus observed candidemia across low- and high-risk strata as demonstrated by insignificant *P *values in both cohorts (all Hosmer-Lemshow chi-squared test *P *> 0.10, a larger *P *value is better, because it suggests that predicted and observed incident rates are in higher agreement across low and high risk stratus). The full model also provided good calibration in the derivation cohort (*P *= 0.74) but not in the validation cohort (*P *= 0.02), suggesting over- or under-prediction in some risk strata when additional variables were added to the model.

## Discussion

Our analysis demonstrates that a simple equal-weight risk stratification score can assess the potential for candidemia in newly hospitalized patients with BSI. We validated our model using a cohort of patients discharged during the two consecutive years after the derivation cohort. The cohorts had similar graded risk of candidemia that increased with increased number of risk factors. The equal-weight risk-score model provided similar between-cohort discrimination for the risk of candidemia and goodness of model fit, indicating the stability of our risk score. In a sensitivity analysis, the equal-weight risk-score model provided nearly identical discrimination and goodness of fit compared with that of unequal-weight model. A full 16-risk factor model provided slightly better discrimination but was less robust. Importantly, the equal-weight risk-score model is easier to apply than the other two models.

The need for a risk stratification scheme is pressing. Although *Candida *may be an infrequent cause of BSI on admission, epidemiologic data indicate that the rate of this is likely to increase. The expansion of healthcare delivery beyond the hospital continues apace, and multiple studies now document the evolution of healthcare-associated infections that are distinct from community-acquired or nosocomial infections [13-15]. The likelihood of an increasing prevalence of candidemia at admission, along with the need to ensure that such patients receive early and appropriate antifungal therapy, underscores the anticipated benefit of easy-to-use risk stratification. Prior efforts at risk stratification for candidemia as a cause of nosocomial BSI have been largely unsuccessful due to the lack of a large clinical data set to model such infrequent events. Our effort builds on earlier analyses [30,31] by focusing on a distinct cohort of patients and by using multiple statistical methods to cross-validate the algorithms. Moreover, many adjuncts to a clinically based risk stratification scheme, such as relying on the colonization index or serodiagnostic testing, are less likely to be available in patients presenting to the hospital.

Our risk-score comprised six demographic, patient history, and clinical findings that are routinely available in any acute-care hospital setting and that were previously shown to be associated with adverse outcomes [8,32]. To minimize the time needed to assess the risk of candidemia, we excluded variables requiring laboratory testing.

Our risk score offers several advantages over previous models [30,31]. First, as noted above, our variables were routinely available at presentation and did not require cultures or other tests to confirm the presence of colonization, sepsis, or other conditions. This increases the scores practical value for rapid assessment of risk for candidemia. Second, the accuracy and robustness of our risk score was supported by derivation from a cohort comprising 64,019 patients and validation from a different cohort comprising 24,685 patients in a different time period. Most previous studies of risk assessment in candidemia did not include any retrospective or prospective validation. Third, our results are likely to be generalizable to a broad range of patients presenting to acute-care hospitals because they are derived from teaching and non-teaching hospitals and from urban and rural hospitals, and are not limited to patients in intensive care units. Fourth, we used the concomitant presence of candidemia code and a positive blood culture to identify candidemia case and included acute clinical presentation on admission as candidate variables, which is likely to be a strength of our paper because many large-scale databases tend to only have the results of administrative coding and lack actual culture confirmation.

Our risk score seems consistent with the pathogenesis of candidemia, which includes: increased fungal burden or colonization, often due to broad-spectrum antibacterial therapy or previous health care exposure; disruption of mucosal and skin barriers, often due to indwelling vascular catheters, surgery, trauma, or chemotherapy-related mucositis; and immune dysfunction, which allows dissemination of fungal colonies [16]. For example, previous admission within 30 days and admission from another health care facility, which were important in our model, are likely represent markers for the first and second steps in the pathogenesis of candidemia. Secondly, the relationship between the need for mechanical ventilation and candidemia has been confirmed by others [32]. Although previous studies found that age was not an independent risk factor for candidemia [30,33], our analyses revealed that among patients with BSIs the younger ones appear potentially more iatrogenically immunosuppressed. For example, patients aged less than 65 years were more likely to be on immunosuppressive therapy (17.0% versus 12.6%; *P *< 0.0001), hemodialysis (4.5% versus 2.7%, *P *< 0.0001), or have metastatic cancer (6.0% versus 4.7%; *P *< 0.0001). Similarly, cachexia was associated with metastatic cancer (6.7% versus 4.9%; *P *< 0.0001), immunosuppressed status, or other severe clinical conditions making patients prone to repeated hospitalization and infections. Furthermore, hypothermia is a risk factor for greater mortality with infection and may suggest that fungal infections are often more severe when detected, or more likely to have a delay in therapy resulting in hypothermia and potentially worse outcomes [34]. In total, our risk score probably captured composite measures for exposures to healthcare delivery and its associated risks for candidemia such as underlying immunosuppression and severity of illness -- both expected risk factors for candidemia. Hence the model appeared robust overall when applied to a separate patient population in a different time period in the validation cohort. The high NPV of a low score indicates that the clinical value of the equal-weight score lies in its ability to identify a group of patients at an exceedingly low risk for candidemia. Given an overall prevalence of 1.2%, which essentially represents the pre-test probability of candidemia in these patients, application of the risk score selects for a group of patients where the risk of candidemia approximates zero. In these subjects antifungal therapy can likely be withheld safely because a low score essentially rules out candidemia. More importantly, this very-low-risk group comprises the bulk of the subjects. Alternatively, although the prevalence of candidemia in the higher-risk groups remains limited, the score at least can serve to remind clinicians to consider candidemia and to weigh the potential for this along with the presence or absence of other clinical factors.

Our model had several limitations. First, the retrospective design needs to be validated in a prospective study. However, only large databases provide a sufficiently large sample to identify enough candidemia cases for multivariable modeling. To address issues related to bias from utilization of *ICD*-9 coding, we required culture evidence of candidemia. Second, we limited our population to patients with candidemia diagnosed within two days of admission. Extending the observation period may have changed our model. Therefore, our findings are not necessarily applicable for suspected nosocomial candidemia. Similarly, we likely missed cases present at admission but not diagnosed until later during hospitalization because cultures are not always obtained upon admission. Third, information was lacking on some specific risk factors for candidemia. For example, we did not have data on whether patients were receiving total parenteral nutrition on admission, had central venous catheters in place, had been exposed to antimicrobial therapy, or had recently undergone surgery [30,31,33,35]. Nevertheless, we included previous hospitalization within 30 days, immunosuppression status, and cachexia as candidate variables, which were likely to be associated with those known risk factors identified in the previous literature. Our score is meant to serve as an adjunct to clinical decision-making, which might incorporate knowledge of all potential risk factors. It is not meant in any way to supplant bedside decision-making. Finally, our analysis focused on subjects presenting to the hospital. Therefore, this score does not necessarily apply in cases of suspected nosocomial candidemia.

## Conclusions

In conclusion, we derived and validated a simple risk-score model that stratifies patients at risk for candidemia, which may help clinicians to rule out candidemia and to shorten the time required to identify patients at increased risk for this disease. It may also help researchers to stratify clinical trial or other outcome studies based on the risk present. Although prospective validation is required, six easy-to-determine characteristics categorize candidemia risks at early hospitalization.

## Key messages

• Candidemia is associated with substantial morbidity and mortality, yet it is difficult to diagnose because of its nonspecific presentation.

• We developed and validated a risk score consisting of six easy-to-determine characteristics on presentation.

• The candidemia risk score differentiates patients from low to high risk in a graded fashion.

• The risk score may aid physicians in ruling out candidemia and in identifying those at high risk for candidemia early in the hospital stay. It may also be useful for stratifying patients in clinical trials or other outcome studies.

## Abbreviations

AMS: altered mental status; AUROC: area under the receiver operating curve; BSI: bloodstream infection; BUN: blood urea nitrogen; GCS: Glasgow Coma Scale; *ICD*-9-CM: *International Classification of Diseases*, Ninth Revision, Clinical Modification; NPV: negative predictive value; RPART: recursive partition.

## Competing interests

AFS and MHK have received grant support from, and served as investigators for and consultants to Astellas Pharma US, Inc., Merck, and Pfizer. YPT, XS, and RSJ are employees of CareFusion. JS is an employee of Astellas Pharma US, Inc. Acknowledged contributors Vikas Gupta, Ed Cox, and Linda Hyde are employees of Cardinal Health.

## Authors' contributions

AFS contributed to study concept and design, analysis and interpretation of data, drafting the manuscript, critical revision of the manuscript for important intellectual content, statistical expertise, obtained funding and study supervision.

YPT contributed to study concept and design, acquisition of data, analysis and interpretation of data, drafting the manuscript, critical revision of the manuscript for important intellectual content, statistical expertise, obtained funding, administrative, technical, or material support and study supervision.

RSJ contributed to study concept and design, acquisition of data, analysis and interpretation of data, drafting the manuscript, critical revision of the manuscript for important intellectual content, statistical expertise, and administrative, technical, or material support

XS contributed to study concept and design, acquisition of data, analysis and interpretation of data, drafting the manuscript, critical revision of the manuscript for important intellectual content, statistical expertise and administrative, technical, or material support.

JS contributed to study concept and design, drafting the manuscript, critical revision of the manuscript for important intellectual content, and obtained funding.

MHK contributed to study concept and design, analysis and interpretation of data, and critical revision of the manuscript for important intellectual content.

## Supplementary Material

Additional file 1 Word file containing a table that lists the detailed patient characteristics by derivation and validation cohort. Click here for file

Additional file 2 Word file containing a table that lists detailed univariate analysis on variables associated with candidemia. Click here for file
