# Supplementary material for: Candidemia on presentation to the hospital: development and validation of a risk score
Source: Crit Care. 2009 Sep 29;13(5):R156. doi: 10.1186/cc8110 (PMC2784380; doi:10.1186/cc8110)
Supplement: Additional file 1 — Word file containing a table that lists the detailed patient characteristics by derivation and validation cohort. [file cc8110-S1.DOC]

**Derivation cohort**

**(n=64,019)**

**Validation cohort**

**(n=24,685)**

***P***

**value**

**Candidemia**

738 (1.2)

321 (1.3)

0.0697

**Mortality**

9664 (15.1)

3173(12.9)

<0.0001

Age

<65 years

19523 (30.5)

8403 (34)

<.0001

Gender

male

29845 (46.6)

12090 (49)

<.0001

Race

White

48523 (75.8)

20680 (83.8)

<.0001

Albumin (g/dL)

? 1.8

2704 (4.2)

1278 (5.2)

<.0001

1.9 - 2.2

3800 (5.9)

1757 (7.1)

<.0001

2.3 - 2.8

10244 (16)

4307 (17.5)

<.0001

Base units

? -5 or > 8

6258 (9.8)

2686 (10.9)

<.0001

Total bilirubin (mg/dL)

> 2.0

6531 (10.2)

2534 (10.3)

0.7748

Glucose (mg/dL)

<=70

1636 (2.6)

1233 (5)

<.0001

K (mEq/dL)

> 5.6

3133 (4.9)

1182 (4.8)

0.515

5.1 - 5.6

3438 (5.4)

1343 (5.4)

0.675

Na (mEq/dL)

> 145

3600 (5.6)

1206 (4.9)

<.0001

BUN (mg/dL)

> 70

6128 (9.6)

2251 (9.1)

0.0391

51-70

6806 (10.6)

2574 (10.4)

0.3793

Arterial pH

? 7.22

2129 (3.3)

978 (4)

<.0001

7.23-7.36

3784 (5.9)

1706 (6.9)

<.0001

> 7.49

2212 (3.5)

680 (2.8)

<.0001

Bands (%)

> 32

5345 (8.4)

1813 (7.4)

<.0001

Platelets (k/mm

3

)

? 115 or > 360

16754 (26.2)

6621 (26.8)

0.0473

WBC (k/mm

3

)

? 4.5

5514 (8.6)

2147 (8.7)

0.6843

> 27.0

5096 (8)

1980 (8)

0.7604

19.2 - 27.0

9694 (15.1)

3720 (15.1)

0.7923

Troponin I >.7 ng/mL or CKMB >5 ng/mL

Yes

5992 (9.4)

1935 (7.8)

<.0001

Arterial (pO2 <51 or >140 mm Hg) or

(O2 <86% or >98%)

Yes

4297 (6.7)

1720 (7)

0.1733

PT INR > 1.3 or PT > 15 sec

Yes

16453 (25.7)

7166 (29)

<.0001

Temperature (F)

? 94

910 (1.4)

257 (1)

<.0001

94.1 - 96

3867 (6)

630 (2.6)

<.0001

96.1 - 98.0

12140 (19)

3893 (15.8)

<.0001

Pulse (/min)

> 126

11657 (18.2)

5260 (21.3)

<.0001

Systolic BP (mm Hg)

? 60

3337 (5.2)

1074 (4.4)

<.0001

61-70

3511 (5.5)

1286 (5.2)

0.1059

71-100

15938 (24.9)

8584 (34.8)

<.0001

Respiration (/min)

> 39

4478 (7)

1524 (6.2)

<.0001

? 9 or 30-39

9472 (14.8)

3579 (14.5)

0.2661

Severe AMS

Yes

6301 (9.8)

2255 (9.1)

0.0014

Congestive heart failure

13854 (21.6)

5834 (23.6)

<.0001

Valvular disease

6083 (9.5)

2813 (11.4)

<.0001

Pulmonary circulation disease

1403 (2.2)

1040 (4.2)

<.0001

Peripheral vascular disease

5712 (8.9)

2375 (9.6)

0.0012

Paralysis

3163 (4.9)

1275 (5.2)

0.1696

Other neurological disorders

8132 (12.7)

3380 (13.7)

<.0001

Chronic pulmonary disease

13846 (21.6)

6015 (24.4)

<.0001

Diabetes w/o chronic complications

16134 (25.2)

6571 (26.6)

<.0001

Diabetes w/ chronic complications

5317 (8.3)

2403 (9.7)

<.0001

Renal failure

8882 (13.9)

6036 (24.5)

<.0001

Liver disease

2804 (4.4)

1271 (5.2)

<.0001

Peptic ulcer Disease x bleeding

62 (0.1)

15 (0.1)

0.102

Lymphoma

1618 (2.5)

583 (2.4)

0.1553

Metastatic cancer

3245 (5.1)

1262 (5.1)

0.791

Solid tumor w/out metastasis

2693 (4.2)

1136 (4.6)

0.0094

Rheumatoid arthritis/collagen vas

2131 (3.3)

832 (3.4)

0.7564

Coagulopathy

6785 (10.6)

3055 (12.4)

<.0001

Obesity

3131 (4.9)

1784 (7.2)

<.0001

Cachexia

4549 (7.1)

2392 (9.7)

<.0001

Fluid and electrolyte disorders

27509 (43)

11771 (47.7)

<.0001

Chronic blood loss anemia

1164 (1.8)

482 (2)

0.1838

Deficiency Anemias

17048 (26.6)

7724 (31.3)

<.0001

Alcohol abuse

1874 (2.9)

805 (3.3)

0.0092

Drug abuse

1103 (1.7)

543 (2.2)

<.0001

Psychoses

2110 (3.3)

985 (4)

<.0001

Depression

5495 (8.6)

2686 (10.9)

<.0001

**Other Key Clinical Finding and composite variables**

Previous Admission <= 30 Days

11215 (17.5)

4603 (18.7)

<.0001

Admitted from other Health Care Facility

12813 (20)

5581 (22.6)

<.0001

Chronic Hemodialysis

§

2077 (3.2)

Current Medicine on Immunosuppression

§

8925 (13.9)

Current Medicine on Insulin

§

9970 (15.6)

Mechanical Ventilation on admission

5864 (9.2)

2695 (10.9)

<.0001

Note: § These three variables were not available for the validation cohorts.

**Patient characteristics by cohort (details)**

**Demographics**

**Laboratory Findings**

**Number of Admission (% of Total)**

**Comorbidities**

**Altered Mental Status**

**Vital Signs**

**Characteristic**
