# Supplementary material for: Candidemia on presentation to the hospital: development and validation of a risk score
Source: Crit Care. 2009 Sep 29;13(5):R156. doi: 10.1186/cc8110 (PMC2784380; doi:10.1186/cc8110)
Supplement: Additional file 2 — Word file containing a table that lists detailed univariate analysis on variables associated with candidemia. [file cc8110-S2.DOC]

Number of Candidemia /

Number of cases in the

Row (%)

*P*

value

Number of

Candidemia /

Number of cases in

the Row (%)

*P*

value

**Candidemia cases / Total cases**

738 / 64019 (1.2)

321/24685 (1.3)

Age

<65 years

331/19523 (1.7)

<.0001

137/8403 (1.6)

0.001

Gender

male

377/29845 (1.3)

0.0145

170/12090 (1.4)

0.1508

Race

White

525/48523 (1.1)

0.003

247/20680 (1.2)

0.0008

Albumin (g/dL)

? 1.8

83/2704 (3.1)

<.0001

40/1278 (3.1)

<.0001

1.9 - 2.2

75/3800 (2.0)

<.0001

45/1757 (2.6)

<.0001

2.3 - 2.8

137/10244 (1.3)

0.0562

85/4307 (2)

<.0001

Base units

? -5 or > 8

120/6258 (1.9)

<.0001

57/2686 (2.1)

<.0001

Total bilirubin (mg/dL)

> 2.0

74/6531 (1.1)

0.8748

34/2534 (1.3)

0.8467

Glucose (mg/dL)

<=70

24/1636 (1.5)

0.2278

31/1233 (2.5)

0.0001

K (mEq/dL)

> 5.6

80/3133 (2.6)

<.0001

34/1182 (2.9)

<.0001

5.1 - 5.6

57/3438 (1.7)

0.0043

10/1343 (0.7)

0.0644

Na (mEq/dL)

> 145

74/3600 (2.1)

<.0001

34/1206 (2.8)

<.0001

BUN (mg/dL)

> 70

109/6128 (1.8)

<.0001

48/2251 (2.1)

0.0003

51-70

98/6806 (1.4)

0.0189

43/2574 (1.7)

0.08

Arterial pH

? 7.22

49/2129 (2.3)

<.0001

24/978 (2.5)

0.0012

7.23-7.36

81/3784 (2.1)

<.0001

37/1706 (2.2)

0.001

> 7.49

23/2212 (1)

0.6124

13/680 (1.9)

0.1537

Bands (%)

> 32

50/5345 (0.9)

0.12

26/1813 (1.4)

0.6021

Platelets (k/mm

3

)

? 115 or > 360

260/16754 (1.6)

<.0001

111/6621 (1.7)

0.0016

WBC (k/mm

3

)

? 4.5

80/5514 (1.5)

0.0301

31/2147 (1.4)

0.5396

> 27.0

54/5096 (1.1)

0.5162

35/1980 (1.8)

0.0557

19.2 - 27.0

118/9694 (1.2)

0.5186

40/3720 (1.1)

0.1882

Troponin I >.7 ng/mL or CKMB >5 ng/mL

Yes

60/5992 (1)

0.2487

24/1935 (1.2)

0.8075

Arterial (pO2 <51 or >140 mm Hg) or

(O2 <86% or >98%)

Yes

77/4297 (1.8)

<.0001

30/1720 (1.7)

0.0922

PT INR > 1.3 or PT > 15 sec

Yes

224/16453 (1.4)

0.0036

117/7166 (1.6)

0.0032

Temperature (F)

? 94

22/910 (2.4)

0.0003

13/257 (5.1)

<.0001

94.1 - 96

56/3867 (1.4)

0.0759

23/630 (3.7)

<.0001

96.1 - 98.0

175/12140 (1.4)

0.0009

63/3893 (1.6)

0.0566

Pulse (/min)

> 126

159/11657 (1.4)

0.0182

85/5260 (1.6)

0.0228

Systolic BP (mm Hg)

? 60

50/3337 (1.5)

0.0548

24/1074 (2.2)

0.0057

61-70

53/3511 (1.5)

0.0417

19/1286 (1.5)

0.5652

71-100

187/15938 (1.2)

0.7795

125/8584 (1.5)

0.115

Respiration (/min)

> 39

65/4478 (1.5)

0.0521

18/1524 (1.2)

0.6709

? 9 or 30-39

113/9472 (1.2)

0.6913

47/3579 (1.3)

0.9423

Severe AMS

Yes

135/6301 (2.1)

<.0001

51/2255 (2.3)

<.0001

Congestive heart failure

158/13854 (1.1)

0.8781

73/5834 (1.3)

0.7048

Valvular disease

67/6083 (1.1)

0.6933

44/2813 (1.6)

0.1895

Pulmonary circulation disease

14/1403 (1)

0.5826

12/1040 (1.2)

0.67

Peripheral vascular disease

65/5712 (1.1)

0.9124

32/2375 (1.3)

0.8316

Paralysis

49/3163 (1.5)

0.0322

27/1275 (2.1)

0.0082

Other neurological disorders

108/8132 (1.3)

0.113

34/3380 (1)

0.1038

Chronic pulmonary disease

164/13846 (1.2)

0.6933

91/6015 (1.5)

0.0944

Diabetes w/o chronic complications

196/16134 (1.2)

0.3933

86/6571 (1.3)

0.9441

Diabetes w/ chronic complications

53/5317 (1)

0.2658

24/2403 (1)

0.1695

Renal failure

120/8882 (1.4)

0.0593

83/6036 (1.4)

0.5556

Liver disease

45/2804 (1.6)

0.0218

22/1271 (1.7)

0.1642

Peptic ulcer Disease x bleeding

0/62 (0)

0.3949

1/15 (6.7)

0.0665

Lymphoma

14/1618 (0.9)

0.2725

6/583 (1)

0.5585

Metastatic cancer

62/3245 (1.9)

<.0001

25/1262 (2)

0.0285

Solid tumor w/out metastasis

41/2693 (1.5)

0.0663

19/1136 (1.7)

0.257

Rheumatoid arthritis/collagen vas

24/2131 (1.1)

0.907

15/832 (1.8)

0.1931

Coagulopathy

113/6785 (1.7)

<.0001

64/3055 (2.1)

<.0001

Obesity

27/3131 (0.9)

0.1185

10/1784 (0.6)

0.0042

Cachexia

123/4549 (2.7)

<.0001

70/2392 (2.9)

<.0001

Fluid and electrolyte disorders

366/27509 (1.3)

0.0003

161/11771 (1.4)

0.3723

Chronic blood loss anemia

24/1164 (2.1)

0.0034

9/482 (1.9)

0.2673

Deficiency Anemias

239/17048 (1.4)

0.0004

109/7724 (1.4)

0.2998

Alcohol abuse

21/1874 (1.1)

0.8946

9/805 (1.1)

0.6424

Drug abuse

20/1103 (1.8)

0.0382

11/543 (2)

0.1314

Psychoses

28/2110 (1.3)

0.4458

14/985 (1.4)

0.7324

Depression

74/5495 (1.3)

0.1591

36/2686 (1.3)

0.8467

**Other Key Clinical Finding and composite variables**

Previous Admission <= 30 Days

276/11215 (2.5)

<.0001

125/4603 (2.7)

<.0001

Admitted from other Health Care Facility

281/12813 (2.2)

<.0001

133/5581 (2.4)

<.0001

Chronic Hemodialysis

§

34/2077 (1.6)

0.0356

Current Medicine on Immunosuppression

§

137/8925 (1.5)

0.0003

Current Medicine on Insulin

§

145/9970 (1.5)

0.0021

Mechanical Ventilation on admission

136/5864 (2.3)

<.0001

84/2695 (3.1)

<.0001

Note: § These three variables were not available for the validation cohorts.

**Univariate analysis of variables associated with candidemia (details)**

**Laboratory Findings**

**Vital Signs**

**Altered Mental Status**

**Comorbidities**

**Characteristic**

Validation Cohort (n=24,685)

**Demographics**

Derivation Cohort (n=60,419)
